# Mr. Warnock, on Adhesive Straps in Ulcers

**Published:** 1808-08

**Authors:** W. Warnock

**Affiliations:** Assistant Surgeon, Royal Navy. Cawsand Bay


### *To the Editors of the Medical and Physical Journal.*

GENTLEMEN,

I Should wish, through the medium of your valuable publication, to call the attention of the Medical World to a practice that is adopted throughout the generality of men of war, fraught in my opinion with the most pernicious consequences. It is not my intention to enter into any controversy on the subject; all my aim is, to call out the observations of some more clever man than I am. The practice I allude to, is the unlimited use of adhesive straps in ulcers. To Mr. Baynton, the so successful practitioner in the treatment of ulcers, after this plan, I give every credit; but I am positive at the time he recommended it so warmly to the notice of Surgeons, it was far from his intentions, that that it should be so universally adopted as it is at present. By far the majority of ulcers you meet with on board of a man of war, partakes of a scorbutic taint, or are generally attached to scorbutic patients; now I am certain, from my experience in the treatment of those sores, that nothing is so bad as to apply straps of adhesive plaster. When the discharge is copi[illegible]us and of bad matter, I am certain, by compressing the part the ulcer is on, you produce an extraordinary degree of inflammation; you compel the matter which Nature designed should find an exit there, to open an abcess in a more depending part; and by that means, you often have both the old, and this ulcer of your own make, communicate with each other, and by the extensiveness of it, you bring on a discharge highly debilitating to the system of a sailor, who has nothing to support this but a little meal and water for his breakfast, and salt beef; the matter is often absorbed to a more vital post, hectic fever ensues, and your patient is carried off. It will be of no avail to argue from the good effects that have resulted by the treatment of ulcer, after Mr. Baynton's manner; on those where your patient's diet is liberal, the solids have not that tendency to run into a state of mortification you will always meet with at sea; at the same time, I am not an avowed enemy to the practice; on the contrary, no other will be crowned with so much success, when it is adopted at the proper time. I am certain if the practice was not so generally adopted, we should not have the painful sight of seeing men " who have rendered the state some service," wandering about the Streets of London without a leg, imploring your assistance, to bestow a trifle on them to enable them to drag out a miserable existence.

I am, &c.

W. WARNOCK.
Assistant Surgeon, Royal Navy.

*Cawsand Bay, June 6, 1808.*

